# Incidental Emotions and Hedonic Forecasting: The Role of (Un)certainty

**DOI:** 10.3389/fpsyg.2020.536376

**Published:** 2020-10-09

**Authors:** Athanasios Polyportis, Flora Kokkinaki, Csilla Horváth, Georgios Christopoulos

**Affiliations:** ^1^Department of Marketing and Communication, Athens University of Economics and Business, Athens, Greece; ^2^Department of Design, Organisation and Strategy, Faculty of Industrial Design Engineering, Delft University of Technology (TU Delft), Delft, Netherlands; ^3^Institute for Management Research, Radboud University, Nijmegen, Netherlands; ^4^Nanyang Business School, Nanyang Technological University, Singapore, Singapore

**Keywords:** certainty–uncertainty, appraisal dimension, predicted utility, hedonic forecasting, appraisal-tendency framework, affective forecasting, incidental emotion

## Abstract

The impact of incidental emotions on decision making is well established. Incidental emotions can be differentiated on several appraisal dimensions, including certainty–uncertainty. The present research investigates the effect of certainty–uncertainty of incidental emotions on hedonic forecasting. The results of four experimental studies indicate that uncertainty-associated incidental emotions, such as fear and hope, compared with certainty emotions, such as anger and happiness, amplify predicted utility. This amplification effect is confirmed for opposite utility types; uncertainty-associated emotions, when compared with their certainty counterparts, lead to an overprediction of positive utilities and to an underprediction of negative utilities. This effect is mediated by the prediction task uncertainty, providing evidence for a carryover process of the incidental emotion. The effect of task uncertainty on predicted utility is, in turn, partly mediated by attention to the task, suggesting that an affective adaptation process lies behind the amplification of forecasts. Taken together, these findings extend the impact of certainty–uncertainty to the context of hedonic forecasting and further corroborate the impact of incidental emotions in judgment and decision making.

## Introduction

Previous research has established the key role of incidental emotions (i.e., emotions that are unrelated to the decision at hand) on decision processes and outcomes (Lerner and Keltner, 2000; Lerner et al., 2004; Peters et al., 2006; Han et al., 2007; Keltner and Lerner, 2010; Västfjäll et al., 2016). The influence of incidental emotions is based on a carryover process from one situation to the next (Bodenhausen, 1993; Lerner and Keltner, 2000). The Appraisal-Tendency Framework (ATF) (Lerner and Keltner, 2000, 2001) explains these carryover effects by linking emotion-specific cognitive appraisal processes with subsequent behavior. According to the ATF, incidental emotional states trigger an implicit tendency to appraise future events in congruence with the central appraisal dimensions that distinguish and differentiate these states (emotion-to-cognition) (Han et al., 2007).

A key aspect differentiating these incidental emotional states refers to the certainty–uncertainty appraisal dimension, defined by the extent to which people understand what is happening and can predict what will happen in the future (Lerner and Keltner, 2000; Smith and Kirby, 2001). For instance, emotions such as anger, happiness, and disgust occur with a sense of certainty, whereas emotions of hope or fear are associated with a sense of uncertainty (Scherer, 1982; Smith and Ellsworth, 1985). Incidental emotions associated with uncertainty have been shown to decrease stereotyping through increased depth of processing (Tiedens and Linton, 2001), to decrease adjustment from self-generated anchors in numerical judgments (Inbar and Gilovich, 2011), to lower optimistic risk perceptions (Lerner and Keltner, 2001), to increase structured ideation in idea generation (Baas et al., 2012), and to increase deliberative processing during a gambling task (Bagneux et al., 2013).

Research has yet to examine the role of incidental emotions and the corresponding cognitive appraisals, such as certainty–uncertainty, in the context of hedonic (otherwise affective) forecasting. Hedonic forecasts are defined as implicit or explicit forecasts of utility that will be experienced at a later time (Kahneman and Snell, 1992; Kahneman and Thaler, 2006). Hedonic forecasting is an essential domain of decision-making because hedonic predictions not only shape subsequent judgment but also define if these events will make people happy, by how much, and for how long (Wilson and Gilbert, 2003). The construal stage of Wilson and Gilbert’s (2003) hedonic forecasting model suggests that forecasters need to create a mental representation of the forecasted event on which to base their judgments (Griffin et al., 1990; Griffin and Ross, 1991). One possible influence during this construal stage is a person’s current affective state (Gilbert and Wilson, 2000). Previous research (Forgas, 1995) has shown that current affective states shape cognitive representations in mood congruent ways. Drawing on the emotion-imbued choice model (Lerner et al., 2015), according to which incidental emotions, as a part of the current affective state, influence subsequent judgment, we extend this rationale and suggest that, in addition to integral emotions (i.e., emotions arising from the judgment or choice at hand; Lerner et al., 2015), incidental emotions can also influence hedonic forecasts, by carrying over to the prediction task (ATF) and influencing prediction outcomes.

In the present research, we focus on the certainty–uncertainty appraisal dimension of incidental emotions and hypothesize that it can shape the outcome of hedonic forecasts. Specifically, we put forward the proposition that incidental emotions associated with uncertainty (e.g., fear) amplify predicted utility compared to emotions associated with certainty appraisals (e.g., anger). We further propose that two mediation mechanisms are responsible for this effect. The hypothesized effect of certainty–uncertainty on predicted utility is mediated by prediction task uncertainty, providing evidence for a carryover process (ATF) of the incidental emotion to the subsequent task (Tiedens and Linton, 2001). The effect of prediction task uncertainty is, in turn, mediated by attention to the task, indicating a process of affective adaptation to uncertainty. The later mediation is in line with the AREA (attend, react, explain, and adapt; Wilson and Gilbert, 2008) model, according to which uncertain emotional situations trigger higher levels of attention, in order to explain and adapt to them. This process might lead to stronger affective reactions during hedonic forecasting and augment the hedonic quality of the future utility. Because people are ordinarily involved in hedonic forecasts of both positive and negative events, we examine if the hypothesized effects can be generalized for both positive (pleasant) and negative (unpleasant) utilities. We expect uncertainty emotions to generally amplify predicted utility and therefore to increase it in the case of positive utilities but to decrease it in the case of negative utilities.

The present article makes two key contributions by (1) unveiling the effect of certainty–uncertainty, as a key appraisal dimension of incidental emotions, on predicted utility during hedonic forecasting, and (2) exploring the underlying mechanisms for the hypothesized effects.

## Incidental Emotions and Uncertainty

In the study of emotions and decision making, two distinct types of emotions have been identified (Lerner et al., 2015): integral and incidental emotions. Integral emotions arise from the judgment or choice at hand, and research has documented their influence on decision making (Loewenstein et al., 2001; Gigerenzer, 2004). Incidental emotions, on the other hand, although unrelated to the decision task, also play a significant role in shaping decisions (Lerner and Keltner, 2000; Lerner and Tiedens, 2006; Han et al., 2007; Yates, 2007; Keltner and Lerner, 2010), judgments about probabilities of upcoming events (Keltner et al., 1993; Goldberg et al., 1999), information processing (Tiedens and Linton, 2001; Bagneux et al., 2013), and risk perceptions (DeSteno et al., 2000; Lerner and Keltner, 2001). The influence of incidental emotions is based on a carryover process from one situation to the next (Bodenhausen, 1993; Lerner and Keltner, 2000), as explained by the ATF (Lerner and Keltner, 2000, 2001).

The ATF builds on Smith and Ellsworth’s (1985) multidimensional appraisal model, which comprises six cognitive dimensions that capture the entire spectrum of emotional experience: valence, certainty, anticipated effort, attentional activity, control, and self-responsibility versus other responsibility. According to the ATF, appraisal tendencies are goal-directed procedures through which emotions influence subsequent judgments (emotion-to-cognition) in line with their appraisals, until the situation that gives rise to the emotion is resolved. Lerner and Keltner (2000) have established that incidental emotions carry over to subsequent decisions that are unrelated to that emotion (Lerner et al., 1998). Thus, each appraisal dimension can lead to appraisal tendencies as perceptual schemata for subsequent judgments. For example, incidental anger triggers high levels of certainty and is accompanied by a tendency to blame individuals even in situations irrelevant to the source of the emotion (Lerner et al., 1998; Goldberg et al., 1999).

Appraisal theorists have established that emotions differ along the certainty–uncertainty dimension (Smith and Ellsworth, 1985; Smith and Kirby, 2001) and identify it as a core aspect of the emotional experience. For instance, hope and fear are characterized by uncertainty appraisal content, whereas anger and happiness are associated with certainty. People commonly experience incidental emotions in their everyday lives, and these emotions may differ significantly along the certainty–uncertainty continuum. Previous research has demonstrated the effects of certainty versus uncertainty-associated emotions on judgments and evaluations. Lerner and Keltner (2001) have shown that incidental anger (certainty-associated emotion) and fear (uncertainty-associated emotion) have opposite effects on risk preferences. Specifically, angry people tend to pursue risk, while fearful people tend to be risk-averse. The authors explain this pattern of effects on the basis of the cognitive appraisals generated by these emotions (Smith and Ellsworth, 1985), because anger is characterized by appraisals of certainty, whereas fear is associated with appraisals of uncertainty (Han et al., 2007).

Tiedens and Linton (2001) have further shown that emotions associated with certainty (disgust, contentment, anger) lead to higher levels of heuristic processing in a range of tasks, including greater reliance on the source of a persuasive message, more stereotyping, and less attention to argument quality, compared to uncertainty emotions (fear, worry, and surprise). They demonstrate that emotions associated with certainty are characterized by a state of confidence, which leads to a reduced motivation to process information thoroughly and thus to a tendency to rely on an increased use of heuristics. Similarly, Bagneux et al. (2013) have found, in the context of a decision task (the Iowa Gambling task), that certainty-associated incidental emotions lead participants to engage in intuitive processing, while uncertainty emotions lead them to engage in deliberative processing. Specifically, the results of two experiments demonstrate that uncertainty-associated emotions (fear and sadness) led participants to disadvantageous decisions compared with certainty-associated emotions (happiness, anger, and disgust). Bagneux et al. (2013) also note that, in their second experiment, sad participants exhibited a set of disadvantageous decisions until the end of the task. They compare this finding with that of a similar study (de Vries et al., 2008) that unveiled a difference between sad and happy participants only in the second block of decisions during the Iowa Gambling Task (IGT). Bagneux et al. (2013) note that this difference can be explained by the fact that the emotion of sadness induced in their study was associated with higher uncertainty compared to the sadness same emotion induced in the de Vries et al. (2008) experiment. Indeed, sadness appears to be malleable along the certainty–uncertainty dimension continuum (Tiedens and Linton, 2001). Although the above studies focus on different tasks and underlying mechanisms, they all indicate that the certainty–uncertainty appraisal of incidental emotions carries over and affects subsequent judgments.

Certainty emotions often lead people to feel “energized” (Frijda et al., 1989), a state of mind that helps people to feel that they can predict and control future outcomes. In contrast, uncertainty emotions can be threatening to the self and can produce a motivational state in which people seek out available methods to entertain and reduce this feeling of uncertainty. Wilson et al. (2005) also refer to the effects of uncertainty as a pleasure paradox: People want to reduce uncertainty about events, but during this process, they may accidentally diminish the pleasure that events bring when uncertainty is prolonged.

Wilson and Gilbert’s (2008) model of affective adaptation (AREA model) proposes that, when people face self-relevant, unexplained events, they attend to them, react emotionally, reach an understanding, and thereby adapt to them. Whereas in the case of well-understood or not self-relevant events, the adaptation requires limited processing, in the case of uncertain, unexplained events, affective adaptation requires heightened levels of attention and generates stronger affective reactions until an adequate explanation or understanding of the event is reached and uncertainty is resolved. Drawing on the AREA model of affective adaptation, Yang et al. (2017, Study 1) had participants consume a sequence of candies. Participants’ happiness with the consumption experience decreased more slowly when they were exposed to the possibility of a negative consumption instance compared to participants who were certain that their experience would be uniformly positive (Yang et al., 2017). The researchers explain that this effect is driven by a favorable uncertainty resolution mechanism. In a similar vein, Kurtz (2018) argues that affective adaptation plays a key role in the study of affective forecasting and proposes the AREA model as a mechanism that can explain the extent to which we adapt to emotional events through sense-making and uncertainty resolution.

Similarly, Bar-Anan et al. (2009) propose the uncertainty intensification hypothesis, according to which uncertainty amplifies emotional reactions by making ongoing “unpleasant events more unpleasant and pleasant events more pleasant” (p.123). Thus, uncertainty amplifies affective reactions to both positive and negative events. However, the affective system cannot separate “true” (integral) feelings from “false” (incidental) feelings and thus treats any currently experienced emotion as a reaction to the currently attended target (Västfjäll et al., 2016). Therefore, even though the above studies manipulate states of integral uncertainty, it seems plausible that uncertainty as a cognitive appraisal dimension of incidental emotions, through its carryover effect, can have similar consequences on emotional experiences.

## Hedonic Forecasting

Hedonic forecasts are defined as implicit or explicit forecasts of utility that will be experienced at a later time (Kahneman and Snell, 1992; Kahneman and Thaler, 2006). Previous research has investigated hedonic (or affective) forecasting (Kahneman and Snell, 1990, 1992; Linville and Fischer, 1991; Baron, 1992; Kahneman, 1994; Snell et al., 1995; Loewenstein and Frederick, 1997; Loewenstein et al., 1997; Loewenstein and Schkade, 1999; Gilbert and Wilson, 2000; Zeelenberg et al., 2000; Gilbert et al., 2002; Buechel et al., 2017; Kurtz, 2018; Dorison et al., 2019) and revealed a number of biases that distort prediction outcomes through their valence, intensity, or duration (overview in Wilson and Gilbert, 2003). In the Kahneman and Snell (1992) experiments, participants made predictions of their future liking for different stimuli (ice cream, yogurt, and short musical pieces). The results showed that participants generally predicted decreased liking for the stimuli, although their actual experience at that future occasion was often increased liking, or reduced disliking. Therefore, participants tended to underpredict future utilities. These results showed that people can be poor judges of their future hedonic states and can make inaccurate forecasts of utility.

Wilson and Gilbert (2003) proposed a construal stage of hedonic forecasting during which people construct a mental representation of the forecasted event. Then people use their immediate hedonic reactions to this simulation (or “prospection”) as predictors of the hedonic reactions that they will probably have when the events they are simulating come true (Gilbert and Wilson, 2007). Although the future hedonic events may unfold in many different ways, this fact seems to escape the average forecaster, leading to potential misconstrual problems (Gilbert et al., 1998; Loewenstein and Schkade, 1999; Wilson and Gilbert, 2003). People usually construct a small number of mental representations of the future event and often fail to understand that their representations of the future are not truthful representations of the objective reality (Griffin and Ross, 1991).

One possible influence on the construal stage is a person’s current affective state (Gilbert and Wilson, 2000). Affective states during hedonic forecasting can shape cognitive representations in mood congruent ways (Forgas, 1995): people in a specific mood construe the mental representation that is affectively congruent with that current mood because of the selective retrieval of and use of the affect congruent information (Bower and Forgas, 2001). In some cases, estimates of future emotional states become “contaminated” by the individual’s current affective state (Wilson and Brekke, 1994; Wilson et al., 2005). For instance, this mental contamination process has been demonstrated by Loewenstein et al. (2003) in the form of the projection bias, a tendency to project current states to future preferences and evaluations.

In a similar vein, Gilbert and Wilson (2009) argue that, even though mental representations should contain only the essential features that define an event and ignore the features that are incidental to it, our failure to preview the incidental features of future events can alter our emotional responses to them. For instance, in the study of Wilson et al. (2000), participants were asked to predict how they would feel the day after their favorite football team won or lost a game. Before the prediction, some participants were asked to preview the incidental features of the event, while other participants were not. The findings showed that the participants who were not asked to preview the incidental features of the event predicted to be very happy if their team won and very unhappy if their team lost. However, those who were asked to preview the incidental characteristics of the event made much more moderate and accurate emotional predictions (Wilson et al., 2000; Gilbert and Wilson, 2009). Other studies have shown that people overestimate how happy they would be if they moved to California (Schkade and Kahneman, 1998) or became wealthy (Kahneman et al., 2006) because their mental representation of these events fails to include incidental features (Wilson and Gilbert, 2003; Gilbert and Wilson, 2009).

Drawing on the emotion-imbued choice model (Lerner et al., 2015), which treats incidental emotions as part of the current affective state, we extend the above rationale and suggest that, as part of the current affective state, incidental emotions can also influence hedonic forecasts, by carrying over to the prediction task and shaping the outcome of the forecasts through an affective adaptation process. From this perspective, the certainty–uncertainty appraisals of incidental emotions can influence hedonic forecasts, as they carry over and influence future hedonic estimates. Gilbert and Wilson (2007) highlight the need to answer the question of whether people can predict the influence of uncertainty on their happiness and well-being. However, little work has been done to explore how distinct appraisal dimensions of incidental emotions, such as certainty–uncertainty, affect the hedonic forecasting process.

## Hypotheses and Conceptual Framework

Previous research (Rick and Loewenstein, 2008; Lerner et al., 2015) indicates that the role of incidental emotions in judgment and decision making presents an important challenge, because they are, by definition, irrelevant to the decision itself. This is, however, what makes their study both intriguing and pertinent. We argue that the certainty–uncertainty appraisal dimension of incidental emotions influences hedonic forecasting. More specifically, we put forward the proposition that incidental emotions associated with uncertainty (e.g., fear) amplify predicted utility compared to emotions associated with certainty appraisals (e.g., anger). This hypothesized effect is explained as a two-step process. First, incidental emotions associated with uncertainty, such as fear and hope, carry over to future situations and link emotion-specific cognitive appraisal processes with subsequent judgment (Lerner and Keltner, 2000; Lerner et al., 2015). Second, after uncertainty emotions carry over, an affective adaptation process is activated; they trigger higher levels of attention in order to explain them (AREA model; Wilson and Gilbert, 2008) and then lead to stronger affective reactions in these unrelated situations, as a counterbalance mechanism to reduce uncertainty and until this uncertainty is explained, adapted to, and resolved (Wilson and Gilbert, 2008). The focus of the present research is on the intensity of hedonic forecasts and specifically on predicted utility. Following the above rationale, it is expected that incidental emotions associated with uncertainty carry over to the prediction task and afterward amplify predicted utility. In contrast, in the case of incidental emotions associated with certainty, such as anger and happiness, affective adaptation is easier as individuals are dominated by a sense of confidence and control (Blascovich and Tomaka, 1996). Therefore, incidental emotions associated with certainty are not expected to have similar effects on predicted utility. To summarize, it is hypothesized that:

Hypothesis 1: Incidental emotions associated with uncertainty amplify predicted utility compared to incidental emotions associated with certainty.

In line with the ATF (Lerner and Keltner, 2000), we suggest that the effect of the certainty–uncertainty appraisal dimension of incidental emotions on hedonic forecasting is the result of appraisal-congruent judgments. Again, through a carryover process, certainty appraisals of incidental emotions influence the degree of certainty one feels in subsequent situations. Tiedens and Linton (2001) have focused on the carryover effect of certainty–uncertainty on a subsequent stereotyping task; the degree of uncertainty during a stereotyping task was found to have a mediating role in the relationship between certainty–uncertainty and stereotype use. Specifically, when participants felt more certain, they tended to resort more to stereotyping. Similarly, we argue that certainty–uncertainty carries over to the perceived uncertainty of the prediction task. Uncertainty-associated incidental emotions may carry over and generate congruent uncertainty appraisals of the prediction task; in other words, people feel less certain about the prediction task. Prediction task uncertainty, in turn, amplifies predicted utility, when compared to certainty emotions. We therefore test the following hypothesis:

Hypothesis 2: Prediction task uncertainty mediates the relationship between the certainty–uncertainty appraisal dimension of incidental emotions and predicted utility.

Wilson and Gilbert (2008) have shown that uncertain emotional states trigger higher levels of attention, in order to adapt to them, and lead to stronger affective reactions. This idea is based on the attention principle (Pessoa et al., 2002; Kahneman et al., 2004), which suggests that events have greater emotional impact when people are attending to them (Wilson and Gilbert, 2008). Because people tend to attend to events for a limited time, the emotional impact of an event is reduced by subsequent events that draw attention away from it (Kahneman et al., 2004). Hence, the levels of attention maintained during hedonic forecasting, as a consequence of the certainty versus uncertainty-associated emotions, can also shape the decision outcome. Uncertainty-associated emotions, by unconsciously carrying over to the prediction task (Lerner et al., 2015), may trigger higher levels of attention and therefore indirectly augment predicted utility. Consequently:

Hypothesis 3: Attention to the task mediates the effect of prediction task uncertainty on predicted utility.

**FIGURE 1:**
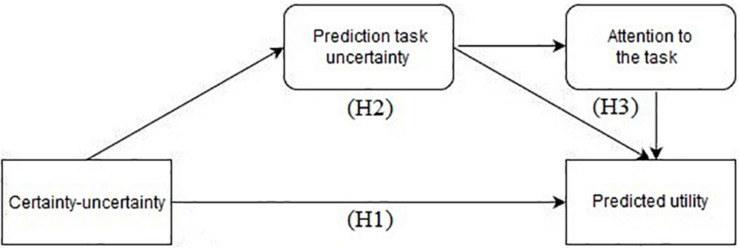
Hypothesized conceptual framework.

The proposed conceptual framework of the present research is illustrated in Figure 1.

## Research Overview

Four experimental studies were set up to test the research hypotheses. Study 1 investigates the effect of the certainty–uncertainty appraisal dimension of incidental emotions on predicted utility by inducing participants to feel either certainty (disgust) or uncertainty (fear) (thereby testing H1). Studies 2, 3, and 4 also test H1 using different manipulations and/or utilities. Study 2, in addition, examines whether this effect (H1) is independent of valence, another key appraisal dimension, and holds for both positive and negative emotions. Study 3 tests H1 and eliminates any possible confounding effects of other appraisal dimensions by manipulating the certainty–uncertainty appraisal dimension of a single emotion (sadness). Study 4 also tests H1 and further examines whether prediction task uncertainty mediates the effect of the certainty–uncertainty appraisal dimension on predicted utility (H2) and whether attention to the task mediates the effect of prediction task uncertainty on predicted utility (H3). In addition, it tests the proposed relations in the context of both positive (pleasant) and negative (unpleasant) utilities, as in Bar-Anan et al. (2009). Emotion induction involved exposure to pretested videos in Study 1 (Han et al., 2012) or an autobiographical emotional memory task in Studies 2, 3, and 4 (Smith and Ellsworth, 1985; Tiedens and Linton, 2001). Following the American Psychological Association Ethical Principles of Psychologists and Code of Conduct (2002, 2016), in all studies we took all the necessary measures to ensure that participants would not be harmed in any way during data collection. Participation was voluntary and participants in all Studies were prompted to immediately withdraw from the experimental session if they felt they would be affected in a negative manner and also that they could terminate their participation at any point during the session. An overview of the studies can be found in the Appendix.

## Study 1

Study 1 examines the hypothesis that incidental emotions associated with uncertainty amplify predicted utility compared to certainty-associated emotions (H1). The study focuses on two emotions of similar (negative) valence in order to control for the effect of valence on hedonic judgments. Specifically, we compared the effect of fear and disgust. Fear is associated with appraisals of uncertainty, whereas disgust is associated with a sense of certainty. These emotions were selected on the basis of relevant literature that documents this difference in their appraisal content (Roseman, 1984; Smith and Ellsworth, 1985). For instance, Tiedens and Linton (2001) have employed disgust and fear as certainty–uncertainty counterparts.

### Materials and Methods

#### Participants and Procedure

Eighty-two postgraduate students (*n* = 82; 62 females, mean_*age*_ = 25.79, *SD* = 1.55 years) participated in the study in exchange for extra course credit. Participation was voluntary. Participants were randomly assigned to one of the two experimental conditions (uncertainty vs. certainty). The data were collected in small group sessions of 8–10 participants. Participants in each session were assigned to the same experimental condition. The manipulation involved exposing participants to a video clip that would induce them to feel either fear (uncertainty) or disgust (certainty). At the beginning of each session, participants were (orally) informed that they would be exposed to material (3-min video clips from well-known films) that might make them experience unpleasant emotions. They were informed that they could withdraw immediately from the study if they felt this would affect them in a negative manner and that they could discontinue their participation at any time. Following the experimental manipulation, participants were offered a stimulus (candy bar) that they were asked to consume. Subsequently, they were led to believe that they would be offered the same stimulus the week after and were asked to respond to the predicted utility measures, as well as to manipulation checks. Finally, participants were exposed to a pleasant video clip to counterbalance the negative emotions they had experienced and were debriefed and dismissed.

#### Manipulation

In order to induce the target emotions, participants in the certainty condition (disgust) were exposed to a selected 3-min video clip from the film Trainspotting (Schaefer et al., 2010; Han et al., 2012), whereas uncertainty (fear) participants watched a 3-min video clip from American Horror Story. The video clips were pretested (see below), and they both contained image and sound and very limited verbal content. Immediately after watching the respective video clip and in order to strengthen the manipulation, participants in both conditions were asked to write down how they would feel if they were in the situation depicted and how they knew that they would feel like that (Smith and Ellsworth, 1985). The main purpose of these questions was to enhance the effectiveness of the manipulation.

##### Pretest

The pretest involved a similar student sample (*n* = 40; 24 females, mean_*age*_ = 25.54, *SD* = 2.10 years). Participants were randomly assigned to watch one of the two videos, as in the main study and were then asked to respond to an adjusted 10-emotion, 5-point, Positive and Negative Affect Schedule (PANAS) scale (Watson et al., 1988). In addition, they were asked to respond to three 11-point (1 = not at all, 11 = very much) items for certainty–uncertainty (Smith and Ellsworth, 1985). Specifically, they rated the degree to which they understood what was happening around them, how well they could predict what would happen next, and how uncertain they were about what was happening when they were feeling the target emotion (α = 0.80). The same measures were used in the main study as manipulation checks. In order to control for possible confounding effects of attentional activity, which is sometimes related with certainty–uncertainty^1^, participants also reported on two 11-point (1 = not at all, 11 = extremely much) items the extent to which they tried to consider this situation further and to devote their attention to it [adapted from Smith and Ellsworth, 1985 (*r* = 0.87)]. Participants in the uncertainty condition reported significantly lower ratings of the certainty appraisal dimension (mean = 4.56, *SD* = 1.47) than their certainty counterparts (mean = 8.13, *SD* = 1.20), *t*(38) = −8.40, *p* < 0.001. Analysis of the PANAS questionnaire for the pretest sample revealed that participants in the uncertainty condition reported significantly more fear (mean = 4.05, *SD* = 0.83) than those in the certainty condition (mean = 2.15, *SD* = 0.81), *t*(38) = 7.34, *p* < 0.001. They also reported significantly less disgust (mean = 2.15, *SD* = 0.88) than their certainty counterparts (mean = 4.15, *SD* = 0.82), *t*(38) = −7.49, *p* < 0.001. There were no significant differences in the ratings of the attentional activity appraisal dimension between the uncertainty (mean = 6.50, *SD* = 2.63) and the certainty (mean = 6.18, *SD* = 2.51) conditions, *t*(38) = 0.40, *p* = 0.69.

#### Measures

##### Predicted utility

Following Kahneman and Snell (1992), participants reported how much they would like and how much they would want the utility in the future consumption occasion (presumably, 1 week later) on 13-point (−6 = dislike very much, 6 = like very much) and (−6 = do not want at all, 6 = want very much) Likert scales, respectively. Predicted utility was operationalized as the mean of these two items (*r* = 0.86).

##### Manipulation checks

The three 11-point (1 = not at all, 11 = extremely much) items, adjusted from Smith and Ellsworth (1985), asking participants to rate the degree to which they understood what was happening around them, how well they could predict what would happen next, and how uncertain they were about what was happening when they were feeling the target emotion (reverse scored), were used for the assessment of the certainty–uncertainty appraisal dimension (α = 0.73). In order to eliminate any confounding effects of attentional activity, participants also reported on two 11-point (1 = not at all, 11 = extremely much) items the extent to which they tried to consider this situation further and to devote their attention to it (adapted from Smith and Ellsworth, 1985), used for the assessment of the attentional activity appraisal dimension (*r* = 0.94). They were also asked to report how intensely they felt each of 10 emotions, adjusted from the PANAS questionnaire (Watson et al., 1988) on 5-point (1 = not at all, 5 = very much) scales.

### Results

#### Manipulation Checks

Two participants were excluded from analysis because of incomplete responses. The manipulation checks indicated that the manipulation of certainty was successful. Participants induced to feel fear reported significantly lower ratings of certainty (mean = 5.43, *SD* = 2.22) than those induced to feel disgust (mean = 8.00, *SD* = 1.27), *t*(78) = −6.18, *p* < 0.001. We further found that the reported rating of disgust was significantly lower in the uncertainty condition (mean = 2.34, *SD* = 0.89) than in the certainty condition (mean = 3.94, *SD* = 0.79), *t*(78) = −8.44, *p* < 0.001. Similarly, the uncertainty participants reported significantly more fear (mean = 3.86, *SD* = 0.88) compared to certainty participants (mean = 1.78, *SD* = 0.80), *t*(78) = 11.01, *p* < 0.001. In addition, we tested for differences in the levels of attentional activity. There was no significant difference on attentional activity between the uncertainty (mean = 6.30, *SD* = 2.93) and the certainty (mean = 5.95, *SD* = 2.67) condition, *t*(78) = 0.53, *p* = 0.60.

#### Predicted Utility

The results revealed a significant effect of certainty–uncertainty on predicted utility. In line with H1, predicted utility in the uncertainty condition was significantly higher (mean = 3.67, *SD* = 1.38) than predicted utility in the certainty condition (mean = 2.17, *SD* = 3.10), *t*(78) = 2.88, *p* = 0.005, Cohen *d* = 0.63.^2^

### Discussion

The results of this study indicate that uncertainty-associated incidental emotions, compared to certainty-associated emotions, amplify predicted utility and therefore provide support for H1. We also show that the hypothesized effect is explained by differences of the emotions induced across certainty–uncertainty and not by differences across the attentional activity appraisal.

Given the negative valence of the emotions employed in this study, we expand our focus in the next study to include both negative and positive valence emotions and thus to further corroborate the effect of certainty–uncertainty appraisals on predicted utility.

## Study 2

Study 1 involved two emotions both of negative valence, fear and disgust. The key aim of Study 2 is to investigate whether the results also hold for positive emotions and are thus independent of the valence of the emotions. In the present study, we focus on anger (certainty) and fear (uncertainty) as negative emotions, and happiness (certainty) and hope (uncertainty) as positive emotions. It should be noted that disgust (Study 1) has been replaced by anger (as a high certainty, negative valence emotion) for two reasons: first, to rule out alternative explanations of our findings, because disgust is, by definition, an emotion with aversive perceptual or sensory qualities (Scherer, 2001), which might interact with the nature of the utility (candy bar); second, because disgust is generally associated with lower attentional activity compared to fear (Smith and Ellsworth, 1985). Although in Study 1 the ratings of attentional activity of induced disgust were similar to those of induced fear, anger and fear are a more representative pair of emotions that share similar attentional activity levels while they are opposite along the certainty–uncertainty appraisal dimension continuum (Smith and Ellsworth, 1985).

### Materials and Methods

#### Participants and Procedure

Eighty postgraduate students (*n* = 80; 50 females, mean_*age*_ = 33.38, *SD* = 2.45 years) participated in the laboratory experiment, in exchange for extra course credit. As in Study 1, participation was voluntary; participants were informed that they could withdraw immediately from the study if they felt this would affect them in a negative way and also that they could discontinue their participation at any time. Participants were randomly assigned to a 2 (uncertainty vs. certainty) × 2 (negative vs. positive valence) between-subjects factorial design. In this, they responded to an autobiographical emotional memory task, as a manipulation method, which would induce them to feel anger (certainty, negative valence), fear (uncertainty, negative valence), happiness (certainty, positive valence), or hope (uncertainty, positive valence). Following this manipulation, participants were offered a stimulus (a small bar of chocolate), which they were asked to consume. Subsequently, they were informed that they would be offered the same stimulus 5 days later and were asked to respond to the predicted utility measures. After completing the manipulation checks, participants were debriefed and dismissed.

#### Manipulation

Manipulation was achieved through an autobiographical emotional event task (Smith and Ellsworth, 1985; Strack et al., 1985; Tiedens and Linton, 2001). Subjects were asked to recall and report an experience or event in which they had felt the target emotion (anger, happiness, fear, and hope, depending on the condition). As part of the instructions, they were asked to keep this experience in mind and to respond to four questions. The purpose of these questions was to encourage the subjects to remember their past experiences as vividly as possible and enhance the effectiveness of the manipulation. For instance, in the fear condition, they received the following instructions:

“Please think of a past situation or event where you felt fear. Picture this situation in your mind. Try and remember as vividly as you can what this past fear situation was like, and what happened to make you feel afraid. When you have this fear situation in your mind, answer the questions below: (1) Describe this past fear situation. What was it like to be afraid in this situation? (2) What happened in this situation to make you feel afraid? (3) How did you know that you were afraid in this situation? (4) What did you do in this situation where you were afraid?”

#### Measures

##### Predicted utility

Participants responded to the same measures of predicted utility (*r* = 0.93) as in Study 1 (Kahneman and Snell, 1992), regarding how much they would like and how much they would want the utility at the future consumption occasion, presumably 5 days later.

##### Manipulation checks

The same three 11-point items (adjusted from Smith and Ellsworth, 1985) used in Study 1 as manipulation checks for the certainty–uncertainty appraisal dimension were also used here (α = 0.81). Specifically, participants rated the degree to which they understood what was happening around them, how well they could predict what would happen next, and how uncertain they were about what was happening (reverse scored) when they were feeling the specific emotion. Two 11-point (1 = not at all, 11 = extremely much) items asking participants to rate the degree to which they considered the situation recalled as pleasant and enjoyable (*r* = 0.98) served as manipulation checks for valence (also from Smith and Ellsworth, 1985). In order to control for possible confounding effects of attentional activity, participants also responded to the two 11-point (1 = not at all, 11 = extremely much) items, asking them to what extent they tried to consider this situation further and to what extent they tried to devote their attention to it (Smith and Ellsworth, 1985) (*r* = 0.70).

### Results

#### Manipulation Checks

A 2 (uncertainty vs. certainty) × 2 (negative vs. positive valence) analysis of variance (ANOVA) revealed that the manipulation of the certainty–uncertainty appraisal dimension was successful [*F*(1,76) = 107.29, *p* < 0.001]. Participants induced to feel uncertainty-associated emotions reported significantly lower ratings of certainty (mean = 4.00, *SD* = 1.63) than those induced to feel certainty-associated emotions (mean = 8.00, *SD* = 1.83). Neither the main effect of valence (*p* = 0.28) nor the interaction effect (*p* = 0.26) were statistically significant.

Similarly, a 2 (uncertainty vs. certainty) × 2 (negative vs. positive valence) ANOVA showed that the valence manipulation was also successful [*F*(1,76) = 523.47, *p* < 0.001]. Participants who were induced to feel negative valence emotions reported significantly lower ratings of valence (mean = 1.68, *SD* = 1.35) than those who were induced to feel positive valence emotions (mean = 10.15, *SD* = 1.92). Neither the main effect of certainty (*p* = 0.21) nor the interaction effect (*p* = 0.60) were statistically significant.

A 2 (uncertainty vs. certainty) × 2 (negative vs. positive valence) ANOVA did not reveal any significant effects on measured attentional activity levels for certainty–uncertainty [*F*(1,76) = 0.57, *p* = 0.45], valence [*F*(1,76) = 1.46, *p* = 0.23], and their interaction [*F*(1,76) = 0.23, *p* = 0.63]. The attentional activity levels of fear (mean = 6.07, *SD* = 2.19) and anger (mean = 6.73, *SD* = 2.00) were not significantly different from those of hope (mean = 6.98, *SD* = 2.43) and happiness (mean = 7.13, *SD* = 2.80).

#### Predicted Utility

**FIGURE 2:**
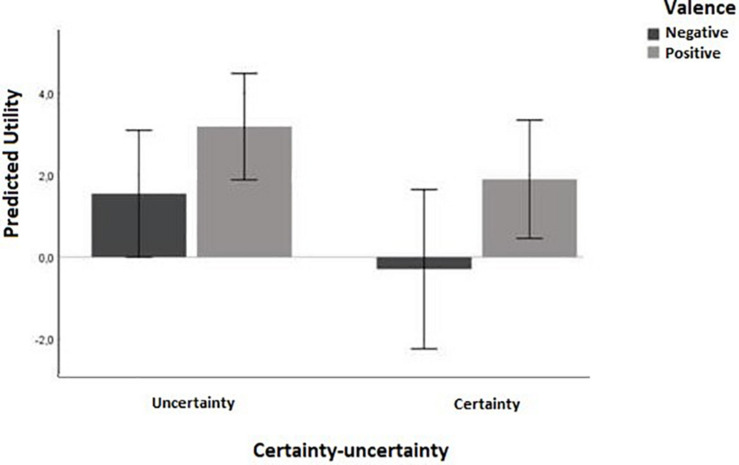
Predicted utility decreases for certainty emotions, for both positive (happiness, hope) and negative (anger, fear) valence.

A 2 (uncertainty vs. certainty) × 2 (negative vs. positive valence) ANOVA revealed a significant main effect of the certainty–uncertainty appraisal dimension on predicted utility [*F*(1,76) = 4.48, *p* = 0.038, η^2^ = 0.06]. Predicted utility in the uncertainty condition was significantly higher (mean = 2.38, *SD* = 3.23) compared to that in the certainty condition (mean = 0.89, *SD* = 3.54) (Figure 2).

The analysis also indicated a significant main effect of the valence appraisal dimension on predicted utility [*F*(1,76) = 6.73, *p* = 0.011, η^2^ = 0.08]. There was no significant interaction between certainty and valence [*F*(1,76) = 0.14, *p* = 0.71].^3^

### Discussion

The findings of the study replicate those of Study 1 and provide additional support for H1 on the effect of certainty–uncertainty appraisals on hedonic forecasting. As in Study 1, uncertainty-associated emotions were found to amplify predicted utility. Moreover, the findings of this study indicate that this effect holds for both positive and negative emotions and is independent of valence. The findings also show that the effect is independent of attentional activity. It should be noted that the valence appraisal dimension had a significant main effect on predicted utility. This can be explained by a mood-congruent mechanism: the valence of the incidental emotion influences the target so that positive incidental emotion makes the evaluation of the target more positive, while negative incidental emotion leads to more negative evaluations (Kahneman, 2003; Västfjäll et al., 2016).

However, the emotions employed here might not only differ in terms of their certainty–uncertainty, valence, or attentional activity appraisals, but might also differ across other appraisal dimensions. In order to further corroborate our first hypothesis and to isolate the effect of certainty–uncertainty, we proceed in the next study to manipulating the certainty–uncertainty appraisal dimension while keeping other appraisal dimensions constant. To achieve this, we focus on a single emotion.

## Study 3

The previous two experiments provide support for the hypothesized effect of the certainty–uncertainty appraisal dimension on hedonic forecasting. A possible criticism and an inherent limitation of Studies 1 and 2 lies on the possibility that this effect is not independent of other appraisal dimensions. This limitation is related to a key methodological issue. In Experiments 1 and 2, the emotions induced differed not only in terms of certainty or uncertainty, but also in terms of other appraisal dimensions, such as anticipated effort and self-responsibility versus other responsibility. In order to control for possible confounding effects, in this study we induce the same emotion and manipulate its certainty–uncertainty appraisals, keeping thus other appraisal dimensions constant. We selected sadness for this study as it is located close to the middle of the certainty–uncertainty dimension (Smith and Ellsworth, 1985). In addition, similar manipulations of the certainty–uncertainty appraisal dimension for the same emotion have been reported in the literature (Tiedens and Linton, 2001).

### Materials and Methods

#### Participants and Procedure

Sixty postgraduate students (*n* = 60; 34 females, mean_*age*_ = 25.77, *SD* = 1.14 years) participated in the study in exchange for extra course credit. As in the previous studies, participation was voluntary, and participants were informed that they could withdraw from the study at any time. Participants were randomly assigned to one of the two experimental conditions (uncertainty vs. certainty). The manipulation was achieved through an autobiographical emotional memory task, as in Study 2. Participants were asked to recall and report a past experience in which they had felt sadness of certainty or uncertainty appraisal content, depending on the condition. Following the manipulation, participants were offered a stimulus (a small chocolate bar) and were informed that they would be offered the same stimulus 1 week later. They were then asked to respond to predicted utility measures and to manipulation checks and were debriefed and dismissed.

#### Manipulation

As in Study 2, an autobiographical emotional memory task (Smith and Ellsworth, 1985; Tiedens and Linton, 2001) was employed for the manipulation of certainty–uncertainty. Participants in the certainty condition were asked to recall and report an experience or event in which they had felt sadness characterized by a certainty appraisal content (i.e., during which they understood why they were sad and could predict what was going to happen next). Similarly, participants in the uncertainty condition were asked to recall and report an event or experience that had generated sadness with an uncertainty content, during which they did not understand why they were sad and could not predict what would happen afterward. In order to create a more vivid recall, participants in both conditions responded to two open-ended questions about how they felt while recalling the specific event. In the uncertainty sadness condition (and certainty sadness condition, respectively), the instructions were the following:

“Please think of a past situation or event where you felt sadness and you did not know (you knew) what was happening neither (and) you could predict what was going to happen next. Picture this situation in your mind. Try and remember as vividly as you can what this past sad situation was like. When you have this situation in your mind, answer the questions below: (1) Describe this past sadness situation. What was it like to be sad in this situation? (2) How did you know that you were sad in this situation?”

As in the previous studies, the purpose of these questions was to encourage participants to recall their past experiences as vividly as possible.

#### Measures

##### Predicted utility

Participants responded to the same two items (Kahneman and Snell, 1992) as in Studies 1 and 2, of how much they would like and how much they would want the utility at the future consumption occasion, 1 week later. Predicted utility was operationalized as the mean of these two items (*r* = 0.73).

##### Manipulation checks

Participants were also asked to respond to the same three certainty items (α = 0.74) as in Study 2 (Smith and Ellsworth, 1985). In order to control for possible confounding effects of attentional activity, participants also responded to the same two items (Smith and Ellsworth, 1985) as in Studies 1 and 2 (*r* = 0.77).

### Results

#### Manipulation Checks

Manipulation checks indicated that the manipulation was successful. Participants in the uncertainty condition reported significantly lower ratings of certainty (mean = 5.01, *SD* = 2.09) than those in the certainty condition (mean = 7.70, *SD* = 1.74), *t*(58) = −5.33, *p* < 0.001. There was no significant difference in the attentional activity levels between the uncertainty (mean = 6.77, *SD* = 2.37) and the certainty (mean = 6.83, *SD* = 2.45) condition, *t*(58) = −0.10, *p* = 0.92.

#### Predicted Utility

The results revealed a significant effect of certainty on predicted utility. Specifically, predicted utility in the uncertainty condition was significantly higher (mean = 4.21, *SD* = 1.55) compared to that in the certainty condition (mean = 3.22, *SD* = 1.73), *t*(58) = 2.33, *p* = 0.023, Cohen *d* = 0.59.^4^

### Discussion

The findings of the study indicate that certainty–uncertainty appraisals have the hypothesized effect on predicted utility even when they vary within the same incidental emotion. These findings offer further evidence on the role of the certainty–uncertainty appraisal dimension in hedonic forecasting and indicate that its effects are independent of other appraisal dimensions such as attentional activity. Having established the effect of certainty–uncertainty appraisals on predicted utility, it is important to unveil the underlying mechanism. In the next study, we focus on the carryover effect of the incidental emotion to the prediction task.

## Study 4

The previous three studies provide converging evidence on the effects of incidental emotions that vary along the certainty–uncertainty dimension on hedonic forecasting. The studies have examined these effects in the case of positive utilities (such as chocolate). Study 4 further explores these effects in the case of negative utilities. In general, a utility function can also have negative values, because the hedonic value of a utility can change substantially and can drop to zero or even become negative, for instance, when consumption continues beyond satiation (Cabanac, 1971). In the present context, negative utility refers to an unpleasant event, an event that is unattractive and people would want to avoid (e.g., watching an unpleasant video clip, Bar-Anan et al., 2009). Because people are involved in hedonic forecasts of both positive and negative events in their everyday lives, we examine if the hypothesized effects can be generalized for both positive and negative utilities. Following H1, we expect uncertainty emotions to generally amplify predicted utility and therefore to increase it in the case of positive utilities but to decrease it in the case of negative utilities.

Furthermore, research has established that the effect of incidental emotions on decision making is a result of appraisal-congruent judgments (Lerner and Keltner, 2000, 2001; Tiedens and Linton, 2001). Certainty–uncertainty appraisals are carried over and influence the degree of certainty or uncertainty one feels in a subsequent decision task. This approach suggests that when people happen to experience an uncertainty-associated emotion, they will tend to feel less certain about the decision task, compared to experiencing a certainty emotion. Following the AREA model (Wilson and Gilbert, 2008), affective adaptation to this emerging decision task uncertainty would require heightened levels of attention and would generate stronger affective reactions. In the case of hedonic forecasting, we therefore expect prediction task uncertainty to mediate the effect of the certainty–uncertainty content of incidental emotions on predicted utility (H2). Following the AREA model, we also expect prediction task uncertainty to determine the level of attention to the task. The level of attention, in turn, is expected to mediate the effect of the prediction task uncertainty on predicted utility (H 3). Importantly, as in Study 2, we focus on anger (high certainty) and fear (low certainty), two emotions with similar attentional activity but opposite certainty–uncertainty appraisal content (Smith and Ellsworth, 1985). In this way, we ensure that the hypothesized effects on predicted utility are not originating from differences in the attentional activity appraisal, but from differential levels of attention to the task (AREA model), as a result of the carryover effect of the certainty vs. uncertainty-associated emotion.

### Materials and Methods

#### Participants and Design

One hundred seven undergraduate students (*n* = 107; 60 females, mean_*age*_ = 20.64, *SD* = 1.01 years) participated in the experiment, in exchange for extra course credit. As in the previous studies, participation was voluntary, and participants were informed that they could withdraw from the study at any time. Participants were randomly assigned to one of four experimental conditions in a 2 (uncertainty vs. certainty) × 2 (negative vs. positive utility type) between-subjects design. The manipulation of the certainty–uncertainty appraisal dimension was accomplished through emotion induction (anger and fear, respectively). As in Studies 2 and 3, incidental emotions were induced through an autobiographical emotional memory task. Subjects were asked to recall and report a past experience in which they had felt anger or fear. Following this manipulation, participants were informed that they would listen to a piece of music. Positive utility condition participants listened to a 3-min version of Pharrell Williams’ “Happy” song. Negative utility participants listened to a 3-min atonal piece of music called “Demons.” Participants in both conditions were informed that the following week they would come again to the laboratory and listen to the same piece of music. They then responded to the predicted utility measures. Subsequently, they were asked to respond to measures of prediction task uncertainty and to measures of attention to the task. They also responded to manipulation checks for the certainty–uncertainty dimension and for utility type (negative vs. positive), and to an adjusted PANAS (Watson et al., 1988) questionnaire. Finally, they were debriefed and dismissed.

#### Manipulation

As in Studies 2 and 3, an autobiographical emotional memory task (Smith and Ellsworth, 1985; Tiedens and Linton, 2001) was used to induce the emotions of anger (certainty) and fear (uncertainty). The same questions used in Study 2 to enhance participants’ recollection of the experience were also used here. Utility type (negative vs. positive) was manipulated by using an unpleasant vs. a pleasant stimulus (piece of music).

#### Measures

##### Predicted utility

As in the previous studies, predicted utility was measured by the two 13-point items (*r* = 0.89) from Kahneman and Snell (1992).

##### Prediction task uncertainty

Participants responded to six items measuring prediction task uncertainty (α = 0.79), adjusted from Kaneko et al. (2018). These items are based on Wilson and Gilbert’s (2008) definition of uncertainty, or the lack of it, according to three aspects of understanding: causes, nature, and implications. Specifically, participants responded to the following items (1 = not at all, 7 = very much): “It was possible to understand why I was asked to predict how much I would like the music next week”; “I was uncertain why I was asked to predict how much I will like the music next week”; “It was possible to describe the prediction task in detail” and “I did not know what the prediction task was about”; “It was possible to predict the consequences of how much I will like the music next week”; and “It was impossible to predict how much I will like the music next week.” The first, third, and fifth items were reverse scored.

##### Attention to the task

Uncertainty toward an emotional event (i.e., a prediction task) prolongs attention to that event (Kaneko et al., 2018). Attention was therefore operationalized in terms of its span. We expected the attention drawn by the decision task to manifest itself in the amount of time participants would remain concentrated on it, even after its completion, and before diverting to some other event. Specifically, participants responded to the following items (1 = not at all, 7 = very much):“After I finished the prediction of how much I will like the music next week, it retained my attention for a long time”; “After my prediction had finished, I thought about it for a long time”; and “After my prediction, I forgot about it immediately.” The third item was reverse scored (α = 0.75; adjusted from Kaneko et al., 2018).

##### Manipulation checks

The three 11-point items (Smith and Ellsworth, 1985) for the certainty–uncertainty appraisal dimension used in the previous studies were also used here (α = 0.74). Similarly, participants were also asked to report how intensely they felt each of 10 emotions in an adjusted 5-point PANAS questionnaire (Watson et al., 1988). The type of utility (negative vs. positive) was operationalized as perceived pleasantness. Participants in each condition were asked to rate the pleasantness (i.e., negative vs. positive) of the respective music track on an 11-point (1 = very unpleasant, 11 = very pleasant) scale.

### Results

#### Manipulation Checks

Seven subjects were excluded from the analysis because of incomplete responses. A 2 (uncertainty vs. certainty) × 2 (negative vs. positive utility) ANOVA indicated that participants who were induced to feel fear reported significantly lower ratings of certainty (mean = 5.44, *SD* = 2.00) than those induced to feel anger [mean = 8.01, *SD* = 1.31, *F*(1,96) = 51.70, *p* < 0.001]. Neither the main effect of the type of utility (i.e., negative vs. positive) (*p* = 0.77) nor the interaction (*p* = 0.46) was statistically significant. The manipulation of the certainty–uncertainty appraisal dimension was therefore successful. In addition, the reported rating of anger for the uncertainty condition participants was significantly lower (mean = 2.72, *SD* = 1.51) than that of the certainty condition participants [mean = 4.08, *SD* = 1.33, *t*(78) = −4.78, *p* < 0.001]. Similarly, uncertainty participants reported significantly more fear (mean = 4.38, *SD* = 0.95) than certainty participants [mean = 1.88, *SD* = 1.11, *t*(98) = 12.07, *p* < 0.001]. The manipulation check of the type (negative vs. positive) of utility was also successful. The positive utility was rated as more pleasant (mean = 8.25, *SD* = 1.08) than the negative utility [mean = 2.26, *SD* = 1.60, *t*(98) = −22.37, *p* < 0.001].

#### Predicted Utility

**FIGURE 3:**
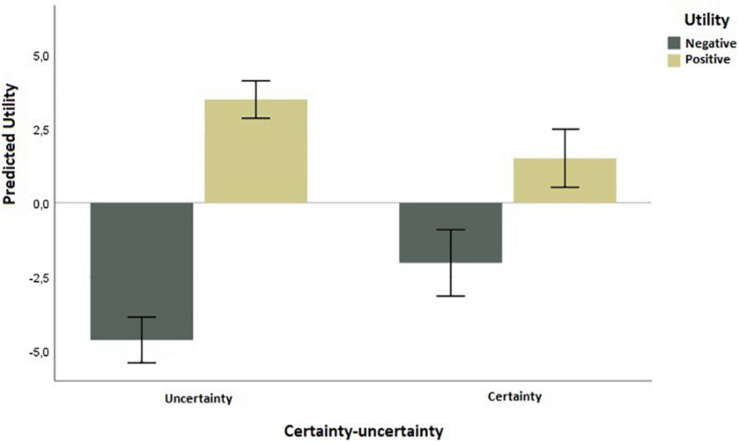
On the interaction effect of certainty–uncertainty and type of utility on predicted utility, for both positive and negative utilities.

To test H1, that uncertainty amplifies predicted utility and therefore increases predicted utility for positive utilities and decreases predicted utility for negative utilities, we performed a 2 (uncertainty vs. certainty) × 2 (negative vs. positive utility) ANOVA. The analysis revealed a significant interaction of the certainty–uncertainty appraisal dimension and utility type [*F*(1,96) = 26.96, *p* < 0.001, η^2^ = 0.22]. Predicted utility in the negative utility, uncertainty condition, was lower (mean = −4.63, *SD* = 1.65) than in the negative utility, certainty condition (mean = −2.03, *SD* = 2.32). Similarly, predicted utility in the positive utility, uncertainty condition was higher (mean = 3.48, *SD* = 1.69) than in the positive utility, certainty condition (mean = 1.50, *SD* = 2.66) (Figure 3).

The main effect of utility (negative vs. positive) was also significant, *F*(1,96) = 173.82, *p* < 0.001. The main effect of certainty was non-significant [*F*(1,96) = 0.48, *p* = 0.49].

A simple effects analysis, using the SPSS GLM syntax command, revealed that uncertainty amplifies predicted utility, for both negative [*F*(1,96) = 14.21, *p* < 0.001] and positive [*F*(1,96) = 12.96, *p* = 0.001] utility types.

#### The Mediating Role of Prediction Task Uncertainty on the Effect of Certainty–Uncertainty on Predicted Utility

To examine the mediating role of prediction task uncertainty on the effect of the certainty–uncertainty appraisal dimension on predicted utility (H2), we split the sample to the two subsamples, depending on the type (negative vs. positive) of utility.

For the positive utility subsample (*n* = 61), we conducted path analysis. Prediction task uncertainty was found to be a mediator of the effect of certainty on predicted utility. Certainty–uncertainty significantly predicted prediction task uncertainty (β = −0.39, *p* = 0.002, 95% confidence interval [CI] [−1.67, −0.40]). We also found that certainty–uncertainty was a significant predictor for predicted utility (β = −0.41, *p* = 0.001, 95% CI [−3.13, −0.84]). Prediction task uncertainty fully mediated (β = 0.91, *p* < 0.001, 95% CI [1.46, 1.85]) the effect of certainty (β = −0.06, *p* = 0.31, 95% CI [−0.78, 0.25]) on predicted utility. The direction of the effects indicates that higher levels of the certainty–uncertainty appraisal dimension (i.e., certainty-associated emotions) led to lower levels of prediction task uncertainty, which in turn contributed to lower predicted utility.

**FIGURE 4:**
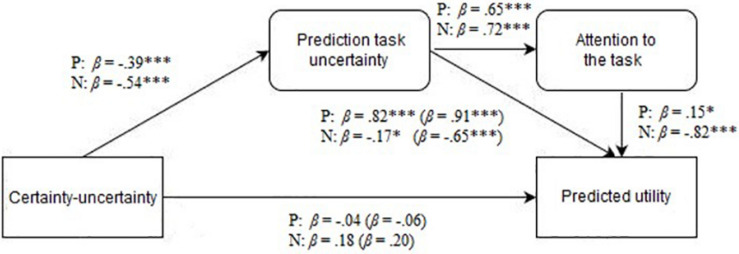
Testing the effect of prediction task uncertainty and attention to the task on predicted utility. β refers to the standardized regression coefficient for the positive (P) and negative (N) utility conditions. The standardized regression coefficients without attention (for prediction task uncertainty) or with prediction task uncertainty and without attention (for certainty–uncertainty) are shown in parentheses. ^∗^*p* < 0.05, ^∗∗^*p* < 0.01, ^∗∗∗^*p* < 0.001.

For the negative utility subsample (*n* = 39), we also conducted path analysis. Prediction task uncertainty was again found to be a mediator of the effect of certainty–uncertainty on predicted utility. Certainty–uncertainty appraisal dimension significantly predicted prediction task uncertainty (β = −0.54, *p* < 0.001, 95% CI [−1.57, −0.49]). We also found that certainty–uncertainty was a predictor for predicted utility (β = 0.56, *p* < 0.001, 95% CI [1.30, 3.99]). Further analysis showed that prediction task uncertainty fully mediated (β = −0.65, *p* < 0.001, 95% CI [−2.20, −0.99]) the effect of certainty–uncertainty (β = 0.20, *p* = 0.10, 95% CI [−0.21, 2.21]) on predicted utility. The results are depicted in Figure 4. The direction of the effects indicates that higher levels of the certainty–uncertainty appraisal dimension (i.e., certainty-associated emotions) led to lower levels of prediction task uncertainty, which in turn contributed to higher predicted utility. Therefore, H2 was supported in both the positive and negative utility conditions.

#### The Mediating Role of Attention to the Task on the Effect of Prediction Task Uncertainty on Predicted Utility

In order to test H3, regarding the mediating role of attention to the task on the relationship between prediction task uncertainty and predicted utility, we conducted path analysis. In the positive utility condition, attention partially mediated (β = 0.15, *p* = 0.016, 95% CI [0.10, 0.93]) the effect of prediction task uncertainty (β = 0.82, *p* < 0.001, 95% CI [1.29, 1.74]) on predicted utility. Furthermore, prediction task uncertainty (β = 0.82, *p* < 0.001, 95% CI [1.26, 1.73]) and attention (β = 0.15, *p* = 0.023, 95% CI [0.07, 0.91]) fully mediated the effect of certainty–uncertainty appraisal dimension (β = −0.04, *p* = 0.47, 95% CI [−0.69, 0.32]) on predicted utility.

Similarly, in the negative utility condition, attention partially mediated (β = −0.82, *p* < 0.001, 95% CI [−1.10, −0.76]) the effect of prediction task uncertainty (β = −0.17, *p* = 0.021, 95% CI [−0.78, −0.06]) on predicted utility. Prediction task uncertainty (β = −0.17, *p* = 0.034, 95% CI [−0.78, −0.33]) and attention (β = −0.82, *p* < 0.001, 95% CI [−1.09, −0.74]) fully mediated the effect (β = 0.18, *p* = 0.79, 95% CI [−0.52, 0.68]) of certainty–uncertainty appraisal dimension on predicted utility. Therefore, H3, regarding the mediating role of attention to the task on the relationship between prediction task uncertainty and predicted utility, was confirmed for both positive and negative utility conditions. The results are depicted in Figure 4.

### Discussion

The results of Study 4 provide support for H1. Uncertainty-associated incidental emotions increase predicted utility for positive utilities, yet they decrease it for negative utilities; thus, an amplification effect of certainty–uncertainty on predicted utility seems to hold for both types of utilities. H2, regarding the mediating role of the prediction task uncertainty, was confirmed for both utility types. These findings further establish that the effect of certainty on predicted utility is a result of the certainty appraisal-congruent judgments and the carryover effect of the certain versus uncertain emotion appraisals to the prediction task (Lerner and Keltner, 2000, 2001). H3 was also confirmed, because attention to the task, as a result of the uncertain versus certain emotion, was found to partially mediate the effect of prediction task uncertainty on predicted utility, for both positive and negative utilities. Hence, the levels of attention maintained, as a consequence of the certainty versus uncertainty-associated emotions, after they carry over to the prediction task, augment predicted utility independently of the utility type.

## General Discussion

### Theoretical Implications

The present research explores the carryover effect of certainty versus uncertainty, as a key appraisal dimension of incidental emotions, on hedonic forecasting. Specifically, the research tests the proposition that uncertainty-associated incidental emotions such as fear or hope augment predicted utility and investigates the underlying mechanism. The findings provide some critical contributions to the study of incidental emotions and their role in judgment and decision-making.

Studies 1, 2, and 3 provide converging evidence that uncertainty emotions amplify predicted utility, compared to emotions with certainty content (H1). Study 1 focuses on two emotions of negative valence, fear and disgust, and shows that fear (associated with uncertainty) increases predicted utility compared to disgust (associated with certainty). Study 2 provides evidence that this effect also holds for positive emotions (hope and happiness) and is thus independent of valence. Fear and hope (associated with uncertainty) were found to amplify predicted utility compared to anger and happiness (associated with certainty).

An inherent limitation of Studies 1 and 2 is related to the probability that the observed effects are not independent of other appraisal dimensions because the focal emotions differ not only in terms of certainty–uncertainty, but also in terms of other appraisal dimensions. To control for possible confounds, we focus, in Study 3, on a single emotion (sadness) and manipulate its certainty–uncertainty appraisal content. The results follow a pattern similar to that of Studies 1 and 2 and further corroborate the effect of certainty–uncertainty on predicted utility. This finding extends the Bar-Anan et al. (2009) uncertainty intensification hypothesis, according to which uncertainty, as an integral emotional state, amplifies affective reactions to both positive and negative ongoing events. We find that uncertainty, as a cognitive appraisal dimension of an incidental emotion, through its carryover effect, has a similar effect on hedonic forecasts.

Moreover, in Study 4, we examine whether this effect holds for both positive (pleasant) and negative (unpleasant) utilities. The findings provide evidence for the amplification effect of certainty–uncertainty on predicted utility; uncertainty emotions, compared to their certainty counterparts, amplify predicted utility regardless of the type of utility (i.e., negative vs. positive). In the case of the positive utility, as in the previous three studies, the uncertainty emotion (fear) was found to increase predicted utility compared to the certainty emotion (anger). In the case of the negative utility, the uncertainty emotion was found to decrease predicted utility; participants predicted that they would dislike the negative utility even more. In addition, in line with the ATF (Lerner and Keltner, 2000, 2001), we have shown that the effect of certainty–uncertainty is the result of appraisal-congruent judgments. Through a carryover process, certainty–uncertainty appraisals of incidental emotions are expected to influence the degree of certainty or uncertainty one feels in the subsequent forecasting task. Study 4 establishes that uncertainty-associated incidental emotions carry over and affect prediction task uncertainty, which, in turn, influences predicted utility. The mediating role of prediction task uncertainty on the link between certainty–uncertainty and predicted utility (H2) is significant for both positive and negative utilities.

Attention to the task, as a result of the uncertain versus uncertain emotion, was found to partially mediate the relation between prediction task uncertainty and predicted utility (H3). This finding is in line with the attention principle (Kahneman et al., 2004), that events have greater emotional impact when people are attending to them. The present findings also extend the AREA model of affective adaptation (Wilson and Gilbert, 2008), because incidental uncertainty also seems to lead to stronger affective reactions and to generate, through a carryover process, responses similar to those of integral uncertainty. In addition, the fact that uncertainty-associated incidental emotions make people overpredict future (positive) utilities indicates that uncertainty may counterbalance and therefore act as a corrective bias toward the generally observed tendency to underpredict future utilities (Kahneman and Snell, 1992).

The findings of the present studies add to previous research that explores the effect of the certainty–uncertainty of incidental emotions on subsequent decisions. This key appraisal dimension has been found to affect structured ideation (Baas et al., 2012), risk perceptions (Lerner and Keltner, 2001), reliance on the expertise of a source of a persuasive message and stereotyping (Tiedens and Linton, 2001), adjustment from self-generated anchors in numerical judgments (Inbar and Gilovich, 2011), and intuitive versus deliberative processing during a gambling task (Bagneux et al., 2013). Our research extends these findings to the important domain of hedonic forecasting and further elucidates the role of this important variable.

### Limitations, Practical Implications, and Future Research

Despite inducing a variety of emotions and using different emotion induction methods, we focused mostly on emotions of negative valence. Only Study 2 examines the hypothesized effects for positive emotions as well. In addition, the present research does not examine possible interaction effects between certainty–uncertainty and other appraisal dimensions. For instance, control, also an important appraisal dimension, might interact with certainty–uncertainty to determine decision outcomes. Future research could employ the manipulations of both certainty–uncertainty and control to examine the interdependence of their impact on decision making. Moreover, a limitation of the present research relates to the absence of a neutral state of uncertainty, which would provide a sort of “baseline” assessment. The highlighted effects indicate that hedonic forecasts become less or more biased because of the certainty versus uncertainty appraisals of the incidental emotions induced. Future research could replicate the present studies by incorporating forecasting error as well, as a dependent variable, and by including a neutral (i.e., not primed for either certainty or uncertainty) state in order to compare the effects of certainty–uncertainty on hedonic forecasts for all three conditions (low, high, and neutral).

The present research may also have practical implications. Incidental emotions are omnipresent in everyday life. Based on prior and the present research, we have seen that incidental emotions of certainty or uncertainty appraisals can affect subsequent decision making. For instance, consumers are influenced by incidental emotions while forecasting purchase decisions. The certainty–uncertainty appraisal dimension of incidental emotions may influence the attractiveness of goods or services in the context of everyday consumer choices. They can influence decisions that everyday people make, such as what to buy, what to wear, or where to travel. Bobko et al. (2013) introduce the concept of consumer suspicion that, as key emotional state, includes an inherent component of uncertainty. A generalized extrapolation of their conceptualization may include incidental uncertainty that, as an everyday consumer phenomenon, can carry over and affect suspicion toward a branded product or an advertising campaign. From this perspective, certainty–uncertainty of incidental emotions might influence predicted utility and therefore alter the attractiveness of products and brands and influence consumer decisions.

Moreover, several important decisions beyond consumer choice require making predictions, be that decisions of governments, companies, judges, or scientists. It seems that incidental states of (un)certainty can actually influence the majority of social decision-making. Future research should shed light on the role of incidental emotions in forecasts for important life events such as whom to marry or which job to apply for (see Rick and Loewenstein, 2008). For instance, Gilbert et al. (1998) studied assistant professors’ predictions of how they would feel after their tenure decisions. Their study revealed that there was actually no difference in well-being between those who had received tenure and those who had not, in either the first 5 or the subsequent 5 years. Future research could examine how the certainty–uncertainty appraisal dimension of incidental emotions can affect the prediction of future important events as the above, instead of everyday utilities, as well as how it can accelerate or prolong affective adaptation to them.

The present research unveils the implications of the certainty–uncertainty appraisal dimension of incidental emotions in the context of hedonic forecasting. Future research needs to corroborate our findings, shed more light on the underlying mechanisms, and explore the interdependence of certainty–uncertainty and other appraisal dimensions.

## Data Availability Statement

The datasets generated for this study are available on request to the corresponding author.

## Ethics Statement

Ethical review and approval was not required for the study on human participants in accordance with the local legislation and institutional requirements. Written informed consent for participation was not required for this study in accordance with the national legislation and the institutional requirements.

## Author Contributions

AP took the lead in conceptualizing the manuscript, data collection, analysis, and writing the manuscript. FK assisted AP with data collection, writing and editing the manuscript. CH and GC contributed to analysis and editing the manuscript. All authors contributed to the article and approved the submitted version.

## Conflict of Interest

The authors declare that the research was conducted in the absence of any commercial or financial relationships that could be construed as a potential conflict of interest.

## Footnotes

1 In the present study as well as in Studies 2 and 3, in addition to measures of certainty–uncertainty, valence and attentional activity, we also collected measures of the remaining appraisal dimensions (control, self-responsibility versus other responsibility and anticipated effort, based on Smith and Ellsworth, 1985). Although these variables are not directly related to our hypotheses, we conducted a series of analyses to test for possible confounding effects. These analyses did not reveal any significant effects and therefore are not reported in the present and the following studies.

2 The data were also analyzed by means of an analysis of covariance (ANCOVA) with certainty–uncertainty as an independent variable, predicted utility as a dependent variable, and attentional activity as a covariate. This analysis confirmed the significant effect of certainty-uncertainty on predicted utility, *F*(1,77) = 8.21, *p* = 0.005, η^2^ = 0.10. Attentional activity was not found to be a significant covariate.

3 The data were also analyzed by means of a 2 (uncertainty vs. certainty) × 2 (negative vs. positive valence) ANCOVA with predicted utility as a dependent variable and attentional activity as a covariate. This analysis confirmed the significant main effect of certainty–uncertainty on predicted utility, *F*(1,75) = 4.50, *p* = 0.037, η^2^ = 0.06. Attentional activity was not found to be a significant covariate.

4 The data were also analyzed by means of an ANCOVA with certainty–uncertainty as an independent variable, predicted utility as a dependent variable, and attentional activity as a covariate. This analysis confirmed the significant effect of certainty–uncertainty on predicted utility, *F*(1,57) = 5.55, *p* = 0.022, η^2^ = 0.09. Attentional activity was not found to be a significant covariate.

## Appendix

**Table:**

**Studies**	**Study 1**	**Study 2**	**Study 3**	**Study 4**

Purpose of study	Test H1	Replicate H1, valence independence	Establish H1 for same emotion	Establish H1, test H2 and H3, type of utility
Sample size	82	80	60	107
Sample	Postgraduate students	Postgraduate students	Postgraduate students	Undergraduate students
Type of study	Laboratory study	Laboratory study	Laboratory study	Laboratory study
Study design	Between-subjects	Between-subjects 2 × 2	Between-subjects	Between-subjects 2 × 2
Excluded from analysis	Two subjects-incomplete responses	None	None	Seven subjects—incomplete responses
IV	Certainty–uncertainty	Certainty–uncertainty, valence	Certainty–uncertainty	Certainty–uncertainty, type of utility
DV	Predicted utility	Predicted utility	Predicted utility	Predicted utility
Emotion induced	Fear, disgust	Anger, fear, happiness, hope	Sadness (UA/CA)	Anger, fear
Induction method	Video	AEMT	AEMT	AEMT
